# Prevalence of stillbirth and associated factors among deliveries attended in health facilities in Southern Ethiopia

**DOI:** 10.1371/journal.pone.0276220

**Published:** 2022-12-13

**Authors:** Jegnaw Wolde, Dereje Haile, Kebreab Paulos, Mihiretu Alemayehu, Asrat Chernet Adeko, Asaminew Ayza

**Affiliations:** 1 Wolaita Zone Health Department, Wolaita Sodo, Ethiopia; 2 Department of Public Health, College of Health and Medical Sciences, Wolaita Sodo University, Sodo, Ethiopia; 3 Department of Midwifery, College of Health and Medical Sciences, Wolaita Sodo University, Sodo, Ethiopia; Tulane National Primate Research Center, UNITED STATES

## Abstract

**Background:**

Stillbirth is an unfavorable outcome of pregnancy, which is still prevalent in many countries despite remarkable efforts made to improve the care of pregnant women. While producing estimates consistent with other national reports, all are hindered by limited data and important causes of death are likely to be missed. However; there is a scarcity of data on stillbirth in Ethiopia particularly in the Wolaita zone.

**Objective:**

To assess the prevalence and associated factors of stillbirth among women giving birth at public hospitals in the Wolaita zone, southern Ethiopia.

**Methods:**

A facility-based cross-sectional study was conducted in public hospitals in the Wolaita zone. A stratified sampling technique was used to select 737 women. A pre-tested interviewer-administered questionnaire was used for data collection. Data were entered using Epidata version 3.1 and analyzed using SPSS version 20. Bivariate and multiple logistic regression analysis were used and the crude and adjusted odds ratios at a 95% confidence interval with P-value <0.05 were considered to declare the result as statistically significant.

**Result:**

This study reported an 8.7% [95% CI: 6.5–10.8] prevalence of stillbirth. Women who lived in rural areas, had pregnancy and labor complications, a high number of pregnancies, a prior history of stillbirth, and a complicated delivery were associated with stillbirth. When compared to urban residents, being a rural resident increased the risk of stillbirth by 2.57 fold [adjusted OR = 2.57, 95% CI: 1.23, 5.40]. When compared to their counterparts, women who experienced complications during pregnancy and labor increased 6.23 fold [AOR = 6.23, 95% CI: 2.67–14.58], having a previous history of stillbirth increased 6.89 fold [AOR = 6.89, 95% CI: 2.57–13.57], and the type of delivery increased 7.13 fold the risk of stillbirth [AOR = 7.13, 95% CI: 2.71–18.73].

**Conclusion and recommendation:**

The prevalence of stillbirth among women who gave birth in public hospitals in the Wolaita zone was found to be high compared to national and regional figures. Therefore, the federal and regional governments should strengthen inter-sectoral collaboration with health facilities to promote the maternal and health care services utilization. The zonal health department and other concerned bodies should focus on the implementation of the strategies and policies that address and reduce the causes of stillbirth.

## Introduction

According to the world health organization’s (WHO) definition, Stillbirth is a baby born with no signs of life at or after 28 weeks of gestation. The burden of stillbirth has remained very high in Sub-Saharan African countries; Ethiopia ranked fifth among the top ten countries with the highest stillbirth rates in the world [1]. Similarly, Ethiopia is the third high-burdened country in east Africa next to Djibouti and Somalia [2]. Efforts to reduce deliveries ending up by stillbirths have not shown much progress in Ethiopia [3].

Stillbirth is an unfavorable outcome of pregnancy, which is still prevalent in many countries despite remarkable efforts to improve the care of pregnant women. It is a neglected tragedy with an estimated 2.6 million deaths per year worldwide and seven thousand women experience a stillbirth a day globally [2]. All most all (99%) occur in low and middle-income countries and 60% occur in rural settings. Several studies in low and middle-income countries [4–6] suggested various determinants for stillbirths. These mainly include lack of accessible obstetric care and inadequate health care service. Furthermore, maternal infections and complications including antepartum vaginal bleeding, pregnancy-related hypertension, failure to give good care during labor and delivery, fetal growth restriction and congenital anomalies and socio-demographic conditions (age, parity, religion, residence, and health service access) are the most important risk factors [5, 6]. In low and middle-income countries, by far the largest source of data on stillbirths comes from population-based household surveys [7]. Although studies have identified several causes of stillbirth, there exists little evidence on the causes of stillbirth in Ethiopia.

The 2016 Ethiopian demographic and health survey (EDHS) showed that the stillbirth rate of Ethiopia was 11.8 per 1000 pregnancies [8]. There is a problem with appropriate data on stillbirth in developing countries such as Ethiopia. While producing estimates consistent with other national reports, all are hindered by limited data and important causes of death are likely to be missed. The data generally available in low and middle-income countries to inform the cause of death often includes only a limited obstetric and/or reproductive history [4]. A recent study from Ethiopia showed low stillbirth rate among women getting prenatal care. Nevertheless, delivery in a health institution did not decrease the chance of having a stillbirth compared with home deliveries [3]. Evidence reported that obstetric emergencies, including antepartum hemorrhage and maternal hypertensive disorders (preeclampsia and eclampsia), are important contributors of stillbirth [9]. Therefore, it is better to improve obstetric and reproductive conditions and other factors such as health service access, behavioral factors, and socio-demographic conditions.

There is limited evidence on factors associated with stillbirth occurring in health facility settings and most of the existing evidences are retrospective surveys [6, 10] which are associated with recall bias. One study by Dejene Tilahun et al. shows the incidence and determinants of stillbirth. This study doesn’t appraise the appropriateness of health service access and doesn’t provide pieces of evidence of behavioral factors. Besides most of the data are not recorded and sources of data very difficult to obtain. As a result, there is no evidence of the magnitude and causes of stillbirth among women who gave birth in public and private hospitals in the study area. Stillbirths are increasingly being counted at a local level; however, the absence of global goals and reporting mechanisms continues to restrict their visibility, especially in the countries with the greatest disease burden. The overall aim of this study was therefore to assess the prevalence and associated factors of stillbirth among women who delivered in public hospitals of Wolaita zone.

## Methods and materials

### Study design and period

A facility-based cross-sectional study design was conducted in health facilities in the Wolaita zone from August 2019 to September 2019.

### Study setting

Wolaita Zone is one of the 14 Zones in southern nations, nationalities, and peoples’ regional(SNNPR) government; at a distance of 380 km from Addis Ababa, the capital city of Ethiopia. This zone consists of 16 Woredas/districts and 6 city administrations. The projected total population was 2,085,727 with (1,022,006) males and (1,063,721) females. There are about 72 health centers, 4 primary hospitals, 2 private general hospitals, and 1 teaching and referral hospital. According to the Wolaita zone health department health management information system (HMIS) report, 2018, stillbirth was 160 per 45582 live births, from which mostly about 95% were reported from public hospitals.

### Population

All deliveries of women in the public and private health facilities of Wolaita Zone was considered as source population. And all deliveries of women in the selected public and private health facilities were taken as the study population.

### Sample size determination

#### 1^st^ objective

The sample size is calculated for the 1^st^ objective by using the single population proportion formula taken from the study done on incidence and determinants of stillbirth among women who gave birth in Jimma University specialized hospital, Ethiopia [6] with the prevalence of 8% of stillbirth.

$$\text{n}=\frac{{\left(\text{Z}1-\alpha /2\right)}^{2}*\text{P}*\left(1-\text{P}\right)}{{\text{d}}^{2}}$$

Where

n = estimated Sample Size

Z1-α/2=the standard normal value corresponding to the desired level of confidence 95% corresponds to the value of 1.96.

d = margin of sampling error tolerated 5% =0.05

P = is an estimate of the prevalence rate for the population (an assumption that stillbirth deliveries among laboring women in the study area)

$$\text{n}=\frac{{\left(1.96\right)}^{2}*0.08*\left(1-0.08\right)}{0.05^{2}}=113$$

So by considering a 5% none—response rate, the total sample required was 119.

#### 2^nd^ objective

The sample size for the second objective is calculated using OpenEpi statistical software version 3.03 for factors associated with stillbirth among delivered women from previous studies (Table 1).

**Table 1:** Sample size determination of 2^nd^objective.

S. no	Variables	CI	Power	% of controls exposed	References	Ratio of controls to cases	AOR	Sample Size	Non-response	Total sample size
1	Absence of complication	95%	80	30.3	17	1:1	0.1	78	4	82
2	Referral from other facility	95%	80	30.3	17	1:1	0.3	166	8	174
3	ANC follow-up	95%	80	2.9	12	1:1	0.03	702	35	737

The sample size calculated for the second objective is higher than the sample size calculated for the first objective. Therefore, the largest sample size 737 is used as the final sample size for this study.

### Sampling techniques

The stratified sampling technique was used to select the study participants. A simple random sampling method was used to select the required health facilities in the Wolaita zone. From 72 health centers, 4 primary hospitals, 2 private general hospitals, and 1 teaching and referral hospital, two primary hospitals (BALE and BITENA primary hospitals) and one referral hospital (WSU teaching and referral hospital), and seven health centers namely Sodo, Bodit, Badessa, Dimtu, Gununo, Gasuba, and Humbo health centers which provides routine delivery services for laboring women were selected randomly and the required sample size was allocated to selected health facilities proportionally.

### Measurement

The structured questionnaire adapted from similar studies was used [11, 12]. It is divided into five parts. The first section inquired about personal data, including age, occupation, ethnicity, religion, and educational level. The second part elicited information about Obstetric and Reproductive history. The third section was Health service access variables. The fourth section inquired about Behavioral history. The fifth part elicited information about Maternal-fetal factors.

### Data collection process

Eight diploma graduate midwifery nurses as data collectors and 1 BSc midwifery and 1 health officer supervisor who fluently speak Amharic and Wolaita language were recruited. The questionnaire was prepared in English and then translated into Amharic and Wolaita language and back-translated to English by language experts to check its consistency. Two days of in-depth training was given for data collectors on the overview of research ethics, data collecting tools, and how to fill out the questionnaire. The interviews were conducted after childbirth and before discharge from the facility.

### Data analysis

Data were edited, coded, and entered into Epidata version 3.1 and exported to SPSS 20 statistical software for analysis. After cleaning data for inconsistencies and missing values in SPSS, descriptive statistics were done. Missing data analysis was conducted by assuming data was missing completely at random (MCAR). Bivariate analysis was done to determine the association between each independent variable and the outcome variable. Before building a bivariable binary logistic regression model, variables significant in other studies and having biological plausibility were selected. In bivariable binary logistic regression, all predictor variables with a p-value of less than 0.25 were identified and entered into a multivariable logistic regression model.

Then a multivariable logistic regression model using a backward stepwise selection method at P value< 0.05 and AOR with 95% CI were used to measure the degree of association between independent variables and the outcome variable. Finally, the result was presented by texts, tables, charts, and figures.

### Data quality control

Two days of in-depth training was given to data collectors. Data collection was supervised by supervisors and the principal investigator. A pretest was conducted on 5% of the sample size in Bedessa Health Center, Wolaita zone, southern Ethiopia. Data were cleaned and checked for completeness daily.

### Ethical considerations

Ethical clearance was secured from the ethical clearance committee of the Wolaita Sodo University, College of Health Science and Medicine. The concerned officials at all levels were informed to get the assurance of the study. The purpose, objectives, and importance of the study were explained and both written and verbal informed consent was secured from each participant. The participant was reassured about the loss of the baby and further advice was given. They were told that documents will be kept confidential and have the right to refuse participation totally at any time if they were not comfortable.

## Results

### The socio-demographic characteristics

Of a total of 737 women, 725 women have successfully answered the survey question, which makes the response rate 98.3%. Of these respondents, 571 (78.8%) were grouped into ages20-34 years. The mean age of the respondents was 26.8 (SD± 4.9). The majority of the study participants were followers of the protestant religion 387 (53.4%) and about 292 (40.3%) of the women were housewives (Table 2).

**Table 2:** Frequency distributions of socio-demographic characteristics of women who gave birth in public hospitals of Wolaita zone, SNNPR, Ethiopia, 2019 (n = 725).

Characteristics (n = 725)		Frequency	Percent
Age of mother	<20	96	13.2
	20–34	571	78.8
	> = 35	58	8.0
Residence	Rural	376	51.9
	Urban	349	48.1
Religion	Orthodox	228	31.4
	Catholic	92	12.7
	Protestant	387	53.4
	Muslim	15	2.1
	Others*	3	0.4
Educational status of women	Unable to write and read	206	28.4
	Primary	226	31.2
	Secondary	176	24.3
	Diploma and above	117	16.1
Occupation	Housewife	292	40.3
	Farmer	44	6.1
	Merchant	106	14.6
	Student	97	13.4
	Daily laborer	30	4.1
	Government employee	156	21.5
Ethnicity	Wolaita	662	91.3
	Amhara	18	2.5
	Gurage	15	2.1
	Others**	30	4.1
Monthly income	<500	110	15.2
	500–1500	235	32.4
	> = 1500	201	27.7
	Don’t know	179	24.7
Age at first pregnancy	<20	185	25.5
	20–24	452	62.3
	> = 25	88	12.2

* = 7^th^ day Adventist

** = Gamo, Gofa, Kambata, Silte

### Obstetric and reproductive characteristics

Of the total study participants, 351 (48.4%) had 2–3 children which are alive and 429 (59.2%) of women had 2–4 pregnancies. The majority of the study participants, 699 (96.4%) were at37-42 weeks of gestational age. The majority of the study participants had ANC follow-up 622 (85.8%) and about 379 (60.9%) of the study participants had four and above ANC visits (Table 3).

**Table 3:** Frequency distributions of obstetric and reproductive characteristics of women who gave birth in public hospitals of Wolaita zone, SNNPR, Ethiopia, 2019 (n = 725).

Characteristics (n = 725)	Category	Frequency	Percent
Number of children ever born (n = 725)	1	230	31.7
	2–3	351	48.4
	4–5	110	15.2
	> = 6	34	4.7
Number of pregnancies(n = 725)	1	215	29.7
	2–4	429	59.2
	> = 5	81	11.1
Gestational age in weeks (n = 725)	<37 weeks	12	1.7
	37–42 weeks	699	96.4
	>42 weeks	14	1.9
ANC visit for current pregnancy (n = 725)	Yes	622	85.8
	No	103	14.2
Number of ANC visits(n = 622)	1	21	3.4
	2–3	222	35.7
	> = 4	379	60.9
Time for first ANC visit (n = 622)	<3	42	6.8
	3–5	420	67.5
	> = 6	160	25.7
Iron/folic during ANC visit for current pregnancy(n = 622)	Yes	552	76.1
	No	70	9.7
Admission during Pregnancy and labor (n = 725)	Yes	95	13.1
	No	630	86.9
Diagnosis (n = 95)	Malaria	45	47.4
	Anemia	16	16.8
	Pregnancy-induced hypertension	20	21.1
	Others	14	14.7
Complications during pregnancy and labor	Yes	93	12.8
	No	632	87.2
Excessive bleeding per vagina (n = 725)	Yes	12	1.7
	No	713	98.3
Labor stay in hours (n = 725)	<12	360	49.7
	> = 12	365	50.3
About current pregnancy (n = 725)	Wanted	595	82.1
	Unwanted	130	17.9

* = urinary tract infection, typhoid fever

### Health service access variables

Of the total study participants, 329 (45.4%) used cars as a mode of transportation to the health facility. The majority of the study participants, 475 (65.5%) of the study participants came to the hospital with a referral. Of the total study participants, 602 (83.0%) reached the health facility in less than 30 minutes (Table 4).

**Table 4:** Frequency distributions of health service access characteristics of deliveries attended in public hospitals in Wolaita zone, SNNPR, Ethiopia, 2019 (N = 725).

Characteristics (N = 725)	Category	Frequency	Percent
Mode of transportation	On foot	249	34.3
	Motorcycle	54	7.4
	Car	329	45.4
	Bajaj(taxi)	93	12.8
Come to hospital	With referral	475	65.5
	Without referral	250	34.5
Time to reach the hospital	<30 minutes	602	83.0
	> = 30 minutes	123	17.0

### Lifestyle behavioral variables

Of the total study participants, 692 (95.4%) had no history of alcohol intake in the past 1year and about 719 (99.2%) of the study participants didn’t currently smoke a cigarette. Almost all of the study participants didn’t chew chat currently 719 (99.2%) (Table 5).

**Table 5:** Frequency distributions of lifestyle behavioral characteristics of women who gave birth in public hospitals of Wolaita zone, SNNPR, Ethiopia, 2019 (n = 725).

Characteristics (n = 725)	Category	Frequency	Percent
Alcohol intake in the past 1 year 1 year	Yes	33	4.6
	No	692	95.4
Currently smoke cigarette	Yes	6	0.8
	No	719	99.2
Chewing chat currently	Yes	6	0.8
	No	719	99.2
Used any herbal medication	Yes	39	5.4
	No	686	94.6

### Maternal and fetal characteristics of deliveries

Of the total study participants, 623 (85.9%) had no history of stillbirth. Most of the study participants 531(73.2%) delivered without complication. About 520 (71.7%) of the study participants gave birth with a spontaneous vaginal delivery as a mode of delivery (Table 6).

**Table 6:** Frequency distributions of maternal and fetal characteristics of women who gave birth in public hospitals of Wolaita zone, SNNPR, Ethiopia, 2019 (n = 725).

Characteristics (n = 725)	Category	Frequency	Percent
History of stillbirth	Yes	102	14.1
	No	623	85.9
Type of delivery	Normal	531	73.2
	Complicated	194	26.8
Mode of delivery	SVD	520	71.7
	CS	111	15.3
	Instrumental	94	13.0
Congenital anomalies	Yes	18	2.5
	No	707	97.5
Fetal presentation	Cephalic	638	88.0
	Breach	54	7.4
	Shoulder	18	2.5
	Others	15	2.1
Cord prolapsed	Yes	48	6.6
	No	677	93.4

* = face presentation, brow presentation, limb presentation

### Prevalence of stillbirth

The overall prevalence of stillbirth was 8.7% with (95% CI: 6.5–10.8) among study participants. Of the total study participants, 63 (8.7%) women experienced stillbirth with a stillbirth rate of 87 per 1000 live birth.

### Factors associated with stillbirth

The multivariable logistic regression analysis was done to control confounders of associations with the outcome variable. After adjusting for several significant covariates associated in the bivariate analysis, residence, complication during pregnancy and labor, history of stillbirth, number of pregnancy, and type of delivery was independently associated factors of stillbirth (Table 7).

**Table 7:** Bivariate and multivariate logistic regression analysis of factors associated with stillbirth in public hospitals of Wolaita zone, SNNPR, Ethiopia, 2019.

Variables	Category	Stillbirth		COR(95% CI)	AOR(95% CI)
		Yes	No		
Residence	Rural	43(10.8)	356(89.2)	1.85(1.22–3.69)**	2.57(1.23–5.40)*
	Urban	20(6.1)	306(93.9)	1	1
Maternal age	<20	6(6.2)	90(93.8)	1	1
	20–34	46(8.1)	525(91.9)	1.31(0.83–1.95)	0.23(0.04–1.34)
	> = 35	11(19)	47(81)	3.51(0.92–6.82)	0.15(0.04–0.55)
Complication	Yes	36(38.7)	57(61.3)	14.15(8.02–24.98)***	6.23(2.67–14.58)***
	No	27(4.3)	605(95.7)	1	1
Referral	With referral	14(5.6)	236(94.4)	0.52(0.28–0.95)	0.65(0.28–1.52)
	Without referral	49(10.3)	426(89.7)	1	1
No of pregnancy	1	11(6.3)	164(93.7)	1	1
	2–4	36(9.6)	340(90.4)	1.58(1.41–5.12)**	3.82(1.17–12.47)*
	> = 5	16(9.2)	158(90.8)	1.51(0.52–5.14)	2.07(0.43–9.86)
History of stillbirth	Yes	29(28.4)	73(71.6)	6.88(3.96–11.95)***	5.91(2.57–13.57)***
	No	34(5.5)	589(94.5)	1	1
Mode of delivery	SVD	18(3.5)	502(96.5)	0.17(0.09–0.36)	1.43(0.51–4.01)
	CS	29(26.1)	82(73.9)	1.72(0.87–3.42)	2.59(0.76–8.81)
	Instrumental	16(17.0)	78(83)	1	1
Type of delivery	Normal	14(2.6)	517(97.4)	1	1
	Complicated	49(25.3)	145(74.7)	12.48(6.70–23.24)***	7.13(2.71–18.73)***

* = P<0.05,

** = P<0.01,

*** = P<0.001

The odds of stillbirth were 2.57 (95% CI: 1.23–5.40) times higher among rural residents when compared to urban residents. The study participants with a complication during pregnancy and labor were associated with stillbirth with (AOR6.23; (95% CI: 2.67–14.58)) compared to those with no complication during pregnancy and labor. This study also revealed that the study participants who had two to four pregnancies were 3.82 times more likely to be associated with stillbirth (AOR (3.82; 95% CI: 1.17–12.47)) compared to those with one pregnancy. Those study participants with a history of stillbirth were 6.89 times associated with stillbirth with AOR (5.91; 95% CI: 2.57–13.57) compared to their counterparts. The odds of stillbirth was 7.13 (95% CI: 2.71–18.73) times higher among complicated deliveries when compared to normal deliveries (Table 7).

## Discussion

This study assessed the prevalence of stillbirth and associated factors among deliveries attended in public hospitals in the Wolaita zone in Southern Ethiopia. Accordingly, the prevalence of stillbirth among women who gave birth in public hospitals in this study was found to be 8.7% (95% CI:6.5–10.8). It also showed that residence, number of pregnancy, complications during pregnancy and labor, history of stillbirth, and type of delivery were independently associated factors of stillbirth among deliveries in this study.

The prevalence of stillbirth in this study showed a comparable proportion with the study conducted in the Amhara region, Ethiopia (8.5%) [6]. Besides, it is in line with the study done in Negest Elene Mohammed general hospital in Hosanna town, South Ethiopia (8.6%) [13]. However, the prevalence in this study was higher than the report of the Ethiopian demographic and health survey, SNNPR (2.0%) [8]. This variation might be due to the difference in the setting in which the study was conducted; the current study was conducted only in a hospital where most of the cases were referred from the peripheral health facility after delays that complicate labor.

In this study, the place of residence is significantly associated with stillbirth. Women from rural residences experienced nearly three times more still births compared to urban women. This is in line with the study done in Ethiopia, Hosanna town, SNNPR, and North Wollo zone, Northeast Ethiopia [2, 13, 14]. This is explained by the fact that women residing in a rural areas may not take action early due to poor health access and limited information about pregnancy, labor, and delivery. Besides, there might be an occurrence of delays among women residing in rural areas.

The number of pregnancies was also significantly associated with stillbirth in the current study. Multigravida women were around four times more likely to have stillbirth compared to women with primigravida. A similar finding was reported in the study conducted in the North Wollo zone and suhul hospital Shire Tigray [14, 15]. It might be also the negligence of women to take health services promptly after the first pregnancy. Besides, it might also be due to poor socio-economic status that leads to the sharing of food with family members.

Complication during pregnancy and labor was also significantly associated with stillbirth. Women with a complication during pregnancy and labor were six times more likely to encounter stillbirth compared to those women without complications. This is in line with the study done in Hosanna town and the North Wollo zone [13, 14]. It could be explained that, if untreated and not mentioned by the mother early, it could end the life of the mother not only the fetus. Failure to recognize signs of complications is one of the barriers to the delay in deciding to seek care [16].

The study also revealed that a history of stillbirth was the factor significantly associated with stillbirth. Women with a history of stillbirth were nearly six times more likely to encounter stillbirth than their counterparts. A similar finding was reported in the study conducted in Northwest Ethiopia showing a history of stillbirth as a predictor of stillbirth [17]. This might be because women with a history of stillbirth regarded as being at high risk of another stillbirth and due to some of the poor obstetric health conditions are recurrent [18].

The type of delivery was also significantly associated with stillbirth. In this study, women with complicated deliveries were seven times more likely to encounter stillbirth compared to those women with normal deliveries. This finding goes in line with the finding of the study conducted in Jimma university specialized hospital [11]. This is explained by the fact that most of the women from lower-level health facilities were referred to hospitals when they get complicated. Also, deliveries other than spontaneous vaginal delivery have a high risk of trauma on a baby that leads to the death of a baby before birth.

### Strength of the study

The strength of this study was that it included primary hospitals and referral hospital. The data were collected by health professionals who were involved in similar studies before.

### Limitation of the study

Some limitations of this study were; firstly, the information used in this study was based on self-report. There may be some bias in the reporting, particularly around some of the sensitive issues that were incorporated, for example, history of stillbirth, congenital anomaly, etc. So that, to address these issues confidentiality was strictly maintained for study participants and the data collectors were oriented on the collection of data. Secondly, age data were exposed to recall bias since the women didn’t know the exact date of last birth and date of marriage.

## Conclusion

The prevalence of stillbirth among women who gave birth in public hospitals in the Wolaita zone was found to be high compared to national and regional figures in the study area. Residence, number of pregnancy, complications during pregnancy and labor, history of stillbirth, and type of delivery were independent factors affecting stillbirth. The result of this study remarks that improvements in the quality of maternal health services require strict attention.

### Recommendation

Eventually, based on our findings, the following recommendations are forwarded:

➢ The federal government and regional government should strengthen communication and discussion with a grass-root level health facility to promote women to use maternal and health care services.
➢ The zonal health department and other concerned bodies should focus on the implementation of the strategies and policies causing stillbirth.
➢ The health professionals in the hospital diagnose and treat the complication during pregnancy and labor early.
➢ Finally, we recommend researchers to emphasize on conducting qualitative researches that identify factors which that may affect stillbirth.

## Supporting information

S1 Data (SAV)

S1 File (DOCX)

## List of abbreviations

JHPIEGO: John Hopkins Program for International Education in Gynecology Obstetrics
ANC: Antenatal Care
HEWS: Health Extension Workers
MCH: Maternal and Child Health
TBA: Traditional Birth Attendant
MMR: Maternal Mortality Ratio
FMOH: Federal Ministry of Health
SDG: Sustainable Development Goal
SNNPR: Southern Nation Nationality of People and Republic

## References

[1] Murray C., Wang H & Fullman N, Global, regional, national, and selected subnational levels of stillbirths, neonatal, infant, and under-5 mortality, 1980–2015:A systematic analysis for the global burden of disease study 2015. Lancet global health, 2016. 388: p. 1725–1774. 10.1016/S0140-6736(16)31575-6 PMC5224696 27733285

[2] Berhie K.A. and Gebresilassie H.G., Logistic regression analysis on the determinants of stillbirth in Ethiopia. Matern Health Neonatol Perinatol, 2016. 2: p. 10. doi: 10.1186/s40748-016-0038-5 27660718 PMC5025573

[3] Lindtjorn B., et al., Reducing stillbirths in Ethiopia: Results of an intervention programme. PLoS One, 2018. 13(5): p. e0197708. doi: 10.1371/journal.pone.0197708 29847607 PMC5976193

[4] Goldenberg R.L., et al., Criteria for assigning cause of death for stillbirths and neonatal deaths in research studies in low-middle income countries. J Matern Fetal Neonatal Med, 2018: p. 1–9. doi: 10.1080/14767058.2017.1419177 30134756 PMC6410364

[5] Welegebriel T.K., Dadi T.L., and Mihrete K.M., Determinants of stillbirth in Bonga General and Mizan Tepi University Teaching Hospitals southwestern Ethiopia, 2016: a case-control study. BMC Res Notes, 2017. 10(1): p. 713. doi: 10.1186/s13104-017-3058-y 29301566 PMC6389129

[6] Lakew D., Tesfaye D., and Mekonnen H., Determinants of stillbirth among women deliveries at Amhara region, Ethiopia. BMC Pregnancy Childbirth, 2017. 17(1): p. 375. doi: 10.1186/s12884-017-1573-4 29132338 PMC5683523

[7] Joy E Lawn MGG, T.M.N., Rubens Craig E, Cynthia Stanton and the GAPPS Review Group, Global report on preterm birth and stillbirth (1 of 7): defi nitions, description of the burden and opportunities to improve data. BMC Pregnancy Childbirth, 2010. 10.1186/1471-2393-10-S1-S1 PMC2841772 20233382

[8] Central Statistical Agency Addis Ababa, E., Ethiopia Demographic and Health Survey. 2016.

[9] Lawn J., Blencowe H, Waiswa P, Amouzou A, Mathers C, Hogan D, et al, Stillbirths: Rates, risk factors, and acceleration towards 2030. Lancet global health, 2016. 387: p. 587–603. 10.1016/S0140-6736(15)00837-5 26794078

[10] Seyom E., et al., Maternal and fetal outcome of pregnancy related hypertension in Mettu Karl Referral Hospital, Ethiopia. J Ovarian Res, 2015. 8: p. 10. doi: 10.1186/s13048-015-0135-5 25824330 PMC4374296

[11] Assefa D.T.T., Incidence and determinants of stillbirth among women who gave birth in Jimma University specialized hospital, Ethiopia Pan African Medical Journal, 2017. doi: 10.11604/pamj.2017.28.299.1269 29721130 PMC5927570

[12] AbdouJammeh S., and Sundby Johanne, StillbirthsinRuralHospitalsinTheGambia:ACross-Sectional RetrospectiveStudy. Obstetrics and Gynecology International 2010.

[13] Abdo RA E.T. a.T.F., Prevalence and associated factors of adverse birth outcomes among women attended maternity ward at Negest Elene Mohammed Memorial general hospital in Hosanna town, SNNPR, Ethiopia. journal of women’s health care, 2016. 5: p. 324.

[14] Asmamaw E, D., B., Belaynew W, Birth outcomes among laboring mothers in selected health facilities of North Wollo Zone, Northeast Ethiopia: A facility based cross sectional study. 2013. V5: p. 1141–50.

[15] Tesfay Adhena A.H., Gebre Geladiwos, Dimtsu Balem, Assessment of magnitude and associated factors of adverse birth outcomes among deliveries at Suhul hospital Shire Tigray, Ethiopia from September, 2015 to February, 2016. Res Rev J Med Sci Technol 6, 2017. 1: p. 1–10.

[16] Bankole A, S.G., Okonofua F, Imarhiagbe C, Hussain R, Wulf D, et al., Birth preparedness and complication readiness: a matrix of shared responsibility. Journal of Clinical Medicine and Research, 2010. 2(4): p. 55–60.

[17] Andargie G., et al., Predictors of perinatal mortality in rural population of Northwest Ethiopia: a prospective longitudinal study. BMC Public Health, 2013. 13: p. 168. doi: 10.1186/1471-2458-13-168 23433304 PMC3599850

[18] Lamont K, S.N., Jones GT, Bhattacharya S, Risk of recurrent stillbirth: systematic review and meta- analysis. BMJ. Published online June 24, 2015. 10.1136/bmj.h3080 26109551

