# Supplementary material for: Prevalence of stillbirth and associated factors among deliveries attended in health facilities in Southern Ethiopia
Source: PLoS One. 2022 Dec 13;17(12):e0276220. doi: 10.1371/journal.pone.0276220 (PMC9746959; doi:10.1371/journal.pone.0276220)
Supplement: S1 File — (DOCX) [file pone.0276220.s002.docx]

## English Version Questionnaire

This questionnaire was prepared to gather information on socioeconomic and demographic profile, obstetric and reproductive, health service access, behavioral, and maternal and fetal factors on stillbirth among mothers attended deliveries in hospitals in Wolaita area, SNNPR.

Part I: Identification particulars

Q001.Questionnaire code______________

Q002. Result of interview: 1. Completed 2. Respondent not available

3. Refused 4. Partially completed

Name of data collector____________ signature _______ Date of interview _____/____/_____ Name of Supervisor _______________signature _______ Checked on, Date ___/___ /________

Part II. Socio – economic and demographic characteristics

| S. No | Questions | Alternatives | Skip to question No |
| --- | --- | --- | --- |
| 101 | Residence | 1**.** Rural  2. Urban |  |
| 102 | Current age in years | …......years |  |
| 103 | Religion | 1.Orthodox  2.protestant  3.catholic  4.muslim  5.others (specify)____ |  |
| 104 | Educational level of the mother | 1.Unable to write and read  2.Primary school  3.Secondary school  4.Diploma and above |  |
| 105 | Occupation of the mother | 1.House wife  2.Farmer  3.Merchant  4.Student  5.Daily laborer  6.Gov’t employee  7.Others(specify)_____ |  |
| 106 | Ethnic origin | 1.Wolaita  2.Amhara  3.Gurage  4.Others(specify)____ |  |
| 107 | Monthly income in Ethiopian Birr | 1……..Birr in a month  2.I don’t know exactly |  |
| 108 | How old are you at first pregnancy? | **………**years |  |

Part III. Obstetric and Reproductive history

| S. No | Questions | Alternatives | Skip to question No |
| --- | --- | --- | --- |
| 201 | How many children do you have? | ……….. |  |
| 202 | How many times have you been pregnant? | ………times |  |
| 203 | Gestational age in weeks | ………weeks |  |
| 204 | Do you have ANC follow up for? | 1.Yes  2.No | Skip to Q209 |
| 205 | Number of antenatal care visits | ……….times |  |
| 206 | When did you started first antenatal care visit? | ……….months |  |
| 207 | Have you taken iron/folic during current pregnancy? | 1.Yes  2.No |  |
| 208 | Is there history of admission during pregnancy? | 1.Yes  2. No | Skip to Q210 |
| 209 | If yes to Q211, what is the diagnosis? | 1.malaria  2.pregnancy induced hypertension  3.amemia |  |
| 210 | Is there excessive bleeding per vagina before the onset of labor? | 1.Yes  2. No |  |
| 211 | Have you been treated for any disease before the onset of labor? | 1.Yes  2.No |  |

| 212 | For how long the labor stayed? | ………..hours |  |
| --- | --- | --- | --- |
| 213 | What do you say about this pregnancy? | 1. Wanted  2. Unwanted |  |
| 214 | At what birth interval do you born the last delivery and index pregnancy? | …………..years |  |
| 215 | Was there any complication during pregnancy and labor? | 1.Yes  2. No |  |

Part IV. Health service access variables

| S. No | Questions | Alternatives | Skip to question No |
| --- | --- | --- | --- |
| 301 | What is the mode of transportation to the health facility? | 1.Walking  2.Motor bicycle  3.Car  4. Bajaj (taxi)  5. Others (Specify)…. |  |
| 302 | How did you come to this hospital? | 1.Referral  2.Without referral |  |
| 303 | At what time do you reach health facility? | ............hours |  |

Part V. Behavioral history

| S. No | Questions | Alternatives | Skip to question No |
| --- | --- | --- | --- |
| 401 | Do you have history of alcohol intake in the past 1 year? | 1.Yes  2.No |  |
| 402 | Do you currently smoke a cigarette? | 1.Yes  2.No |  |
| 403 | Do you chew chat currently? | 1.Yes  2.No |  |
| 404 | Have you used any herbal medication during current pregnancy? | 1.Yes  2.No |  |

Part VI. Maternal-fetal factors

| S. No | Questions | Alternatives | Skip to question No |
| --- | --- | --- | --- |
| 501 | Do you have history of stillbirth ever? | 1.Yes  2.No |  |
| 502 | Was the type of delivery normal or complicated? | 1.Normal  2.Complicated |  |
| 503 | By which mode of delivery the mother delivered? | 1.SVD  2.Cesarean section  3.Instrumental delivery |  |
| 504 | Stillbirth for current delivery? | 1.Yes  2. No |  |
| 505 | Congenital anomalyof baby present? | 1.Yes  2.No | Check medical record |
| 506 | Type of fetal presentation | 1.Cephalic  2. Breach  3.Shoulder  4. Others (Specify)……. | Check medical record |
| 507 | Is there cord prolapsed? | 1.Yes  2. No | Check medical record |

End of the interview. Thank you very much!

Annex III: General information sheet and informed consent (Amharic version)

ወላይታ ሶዶዩኒቨርሲቲ ድህረምረቃ ትምህርት ቤት የህብረተሰብ ጤናና የህክምና ኮሌጅ ይህ መጠይቅ በደቡብ ክልል በወላይታ ዞን ውስጥ ባሉ ሆስፒታል ሞቶ የሚወለዱ ህፃናት ስርጭት መጠንና ተያያዥ ምክንያቶችን ለማወቅ የተዘጋጀ ጥናት ነው፡፡

ክፍል አንድ፡ አጠቃላይ መረጃና የስምምነት መጠየቂያ ቅፅ

እንደምን አደሩ/ዋሉ? እኔ አቶ/ወ/ሮ/ሪት ------------------------------------እባላለሁ፡፡ እዚህ የመጣሁት በወላይታ ሶዶ ዩኒቨርሲቲ በህብረተሰብ ጤና ሁለተኛ ድግሪ እየተማሩ ያሉ የአቶ ጀግናው ወልዴ መረጃ ሰብሳቢ ቡድን አባል ሆኜ መጥችያለሁ፡፡ ከዚህ በመቀጠል ሞቶ የሚወለዱ ህፃናትን በተመለከተ የተወሰኑ ቀላል ጥያቄዎችን ልጠይቆት እወዳለሁ፡፡ ከእርስዎ የሚገኝ መልስ የእናቶችንና የህፃናትን ጤና አገልግሎት ለማሻሻል ከፍተኛ እገዛ ይኖረዋል፡፡ ከእርስዎ የሚናገኘውን ማነኛውንም መልስ በምስጥር እንጠብቀዋለን፡፡ ከዚህ ጥናት ጋር በተያያዘ በማነኛውም ቦታ ስምዎ እንደማይመዘገብና እንደማይጠቀስ ልንገልጽዎት እንወዳለን፡፡ በነዚህ ጥያቄዎች ከአርስዎ ጋር ብበዛ 30 ደቂቃ እንቆያለን፡፡ ለሚያደርጉልን ትብብር አስቀድመን ከልብ እናመሰግናለን፡፡ለተጨማሪ መረጃ የጥናቱ ዋና ባለቤት አቶ ጀግናው ወልዴ በሚቀጥሉት አድራሻ ማግኘት ይችላሉ፡፡

ስልክ፡ 0910310782

ኢ-ሜይል፡ jeginawwolde@gmail.com

የስምምነት ዉል ከገለፃ በኃላ

እኔ እንደ አንድ ግለሰብ በጥናቱ ላይ ለመሳተፍ ተጠይቅያለሁ፡፡ በዋናዉ ጥናቱ ባለቤት እንደተገለጸልኝ ከእኔ ጋር የሚደረገዉ የቃለ-ምልልሱ ዋና ዓላማዉን ተረድችያለሁ፡፡ስለዚህ በምርምር ጥናቱ ለመሳተፍ መወሰነን እንደምከተለዉ አረጋግጣለሁ፡፡በመጠይቁ ለመሳተፍ ፍቃደኛ ነዎት?

1.አዎን ፡ በማስመስገን ቃለመጠይቁን መቀጠል

2.አይደለሁም ፡ በማስመስገን ወደ ሌላ ተሳታፊ መሻገር

**Annex IV: Amharic version questionnaire**

የቃለ - መጠይቅና መለኪያዎች ቅፅ

ይህ መጠይቅ በወላይታ ውስጥ ባሉ ሆስፒታሎች ሞቶ የሚወለዱ ህፃናትን በተመለከተ ከወሊድ በኃላ እና የወላድ ክፍል ከመዉጣታቸዉ በፊትለእናቶች የቀረበ ቃለ መጠይቅ ነው፡፡

መረጃ ሰብሳቢ ስም --------------------- ፊርማ------- መረጃ የተሞላበት ሰዓት -----------------

የተቆጣጣሪ ስም --------------------- ፊርማ ------- መረጃ የተጣራበት ሰዓት ---------------

የቃለ-መጠይቅ ቅፅ ኮድ-----------------

ክፍል ሁለት፡ ማህበራዊ፣ኢኮኖሚያዊ እና ስነ ህዝባዊ ገጽታዎችን በሚመለከት

| ተ.ቁ | ጥያቄ | አማራጭ መልሶች | መልስዎ አይደለም ከሆነ ወደ ተ.ቁ ይለፉ |
| --- | --- | --- | --- |
| 101 | የት ነዉ ምኖሩት ? | 1**.** ገጠር  2. ከተማ |  |
| 102 | አሁን እድሜዎ ስንት ነው? | …......ዓመት |  |
| 103 | የተኛው ሀይማኖት ተከታይ ኖት? | 1. ኦርቶዶክስ  2. ካቶሊክ  3. ፕሮተስታንት  4. ሙሲሊም  5.ሌላ(ይገለጽ)___________ |  |
| 104 | የትምህርት ደረጃ ይገልጹልኛል? | 1. መጻፍና ማንበብ የማትችል  2. የመጀመሪያ ደረጃ ያጠናቀቀች  3. ሁለተኛ ደረጃ ያጠናቀቀች  4. ሰርትፊኬት እና ከዛ በላይ |  |
| 105 | ሥራዎ ምንድነው? | 1. የቤት እመቤት  2. አርሶ አደር  3. ነጋዴ  4. ተማሪ  5. የቀን ሠራተኛ  6. የመንግስት ሠራተኛ  7. ሌላ (ይገለጽ) ___ |  |
| 106 | የተኛው ብሔር/ብሔርሰብ ተወላጅ ኖት? | 1.ወላይታ  2. አማራ  3.ጉራጌ  4.ሌላ (ይገለጽ)____ |  |
| 107 | የወር ገቢዎ በግምት ስንት ነው? | 1……..ብር  2. በውል አላውቅም |  |
| 108 | በመጀመሪያ እርግዝና ጊዜ እድመዎ ስንት ነዉ? | 1**………**ዓመት  2.አላስታዉስም |  |

ክፍል ሦስት፡ ጽንስ እና ስነ ተዋልዶ ሁኔታን በተመለከተ

| ተ.ቁ | ጥያቄ | አማራጮች | መልስዎ አይደለም ከሆነ ወደ ተ.ቁ ይለፉ |
| --- | --- | --- | --- |
| 201 | ስንት ልጆች አሉዎ? | 1. ልጅ የለኝም  2. አንድ  3. ሁለት እና ከዛ በላይ |  |
| 202 | እስካሁን ስንት ጊዜ አርግዘዋል? | 1.አንዴ  2.ሁለቴ  3.ሶስት እና ከዛ በላይ |  |
| 203 | የጽንሱ ዕድሜ በሣምንት | ………ሣምንት |  |
| 204 | የቅድመ ወሊድ ክትትል አድርገዋል? | 1.አዎ  2.አይደለም | 208 |
| 205 | ስንት ጊዜ ጉቭኝት አድርገዋል? | 1.አንዴ  2.ሁለት-ሶስት ጊዜ  3.አራት እና ከዛ በላይ |  |
| 206 | የመጀመሪያዉን ጉቭኝት በስንተኛዉ ወር ላይ ነዉ ያደረግሽዉ? | 1.ከአራት ወር በፊት  2.በአራተኛዉ ወር  3.በአምስተኛዉ ወር  4.በስድስተኛዉ እና ከዛ በላይ |  |
| 207 | በቅድመ ወሊድ ክትትል ጊዜ የብረት እንክብል ወስደዋል? | 1.አዎ  2.አይደለም |  |
| 208 | እርግዝናዉ ከተከሰተ በኃላ ለህክምና ተኝተዉ ነበሩ? | 1.አዎ  2.አይደለም | 210 |
| 209 | መልሶዎ ለጥያቄ ቁጥር 211 አዎ ከሆነ፣የተገኘዉ በሽታ ምንድነዉ? | 1.ወባ  2.ደም ማነስ  3.በእርግዝና ጊዜ የሚከሰት ደም ግፊት |  |
| 210 | ምጥ ከመጀመሪዎ በፊት ከማህፀን ከፍተኛ ደም መፍሰስ ነበሪዎት? | 1.አዎ  2. አይደለም |  |
| 211 | ምጥ ከመጀመሪዎ በፊት ለማንኛዉም በሽታ ታክሞዎ ነበር? | 1.አዎ  2.አይደለም |  |

| 212 | ምጡ ለምን ያክል ጊዜ ነዉ የቆየዉ? | 1.< አስራ ሁለት ሰዓት  2.> አስራ ሁለት ሰዓት |  |
| --- | --- | --- | --- |
| 213 | ስለእርግዝናሽ ምን ትያለሽ? | 1.አቅጄ እና ፈልገ ነዉ  2.አላቀድኩም ግን ፈልገዋለሁ  3.አላቀድኩም አልፈለኩም |  |
| 214 | የመጨረሻዉ ወሊድ እና የመጀመሪያዉ እርግዝና በስንት ዓመት ይለያያሉ? | 1.ከሶስት ዓመት በታች  2.ሶስት-አራት ዓመት  3.አምስት ዓመት እና ከዛ በላይ |  |
| 215 | በእርግዝና እና ምጥ ጊዜ ማንኛዉም ዓይነት መወሳሰብ አጋጥሞሻል? | 1.አዎ  2.አይደለም |  |

ክፍል አራት. ባህሪን በተመለከተ

| ተ.ቁ | ጥያቄ | አማራጮች | መልስዎ አይደለም ከሆነ ወደ ተ.ቁ ይለፉ |
| --- | --- | --- | --- |
| 401 | ባለፋው አንድ ዓመት ውስጥ አልኮል ጠጥተዉ ያዉቃሉ? | 1.አዎ  2.አይደለም |  |
| 402 | አሁን ስጋራ ያጨሳሉ? | 1.አዎ  2.አይደለም |  |
| 403 | አሁን ጫት ቅመዉ ያቅማሉ? | 1.አዎ  2.አይደለም |  |
| 404 | በዚህኛው እርግዝና ባህላዊ መድኃኒት ተጠቅመዋል? | 1.አዎ  2.አይደለም |  |

ክፍል አምስት. የእናትንና የህፃንን ሁኔታ በተመለከተ

| ተ.ቁ | ጥያቄ | አማራጮች | መልስዎ አይደለም ከሆነ ወደ ተ.ቁ ይለፉ |
| --- | --- | --- | --- |
| 501 | በታርክ ሞቶ የተወለደ ህፃን አጋጥሞሻል? | 1.አዎ  2.አይደለም |  |
| 502 | የወሊዱ ሁኔታ የተወሳሰበ ወይም ያልተወሳሰበ ነዉ? | 1.ያልተወሳሰበ  2.የተወሳሰበ |  |
| 503 | ወሊድ የተካሄደበት መንገድ? | 1.ያለመሳሪያ እገዛ/ አምጣ  2.በኦፕረሽን  3.በመሳሪያ እገዛ |  |
| 504 | ሞቶ የተወለደ ህፃን ነው? | 1.አዎ  2.አይደለም |  |
| 505 | የተፈጥሮ ችግር ነበረዉ? | 1.አዎ  2.አይደለም | ከህክምና መዝገብ |
| 506 | የሽሉ አቀማመጥ እንደት ነበር | 1.በጭንቅላት  2.በቅጥ  3.በትከሻ  4.ሌላ (ይገለጽ)……. | ከህክምና መዝገብ |
| 507 | እምብርት ቀድሞ ወቶ ነበር? | 1.አዎ  2. አይደለም | ከህክምና መዝገብ |

የመጠይቁ መጨረሻ በጣም አመሰግኖታለሁ!
