# Supplementary material for: Prevalence of stillbirth and associated factors among deliveries attended in health facilities in Southern Ethiopia
Source: PLoS One. 2022 Dec 13;17(12):e0276220. doi: 10.1371/journal.pone.0276220 (PMC9746959; doi:10.1371/journal.pone.0276220)
Supplement: Attachment — Submitted filename: Response to Reviwers.docx [file pone.0276220.s003.docx]

Review Comments to the Author

Reviewer #1: First of all, I thank you for inviting to review manuscript "Prevalence of Stillbirth and associated factors among womens’ deliveries attended in Health Facilities in Southern Ethiopia (PONE-D-21-20973)" for the journal of PLOS ONE. And I have the following comments for the authors that the manuscript might benefit from.
General comments:
- There are so many grammatical and punctuation errors in the whole document and it needs language edition.

Response: thank you for your comment; we have corrected it in the revised manuscript.

- Abbreviations should state with it’s full at the first time and this is not in your work and also have no found in the list of abbreviation section (E.g.: SNNPR).

Response: thank you for your suggestion, we have corrected and highlighted it in the whole documents of the revised manuscript.

- Tables are not in a standard format.

Response: thank you for your comment, we have corrected it.

- Some of your statements have no source (for instance, According to the WHO definition, Stillbirth is a baby born with no signs of life at or after 28 weeks’ gestation) and many more.

Response: we have added the citation.

State/region OR considering the chronological order of references).◊ country◊continent◊- The organization of introduction is not appropriate (it is better organized either worldwide

Response: we have rearranged the introduction section according to your suggestion.

**Abstract:**
- The abstract section is not well stated, for instance, it is better that the background and objective should state with two or three lines as one paragraph.

Response: we have corrected and highlighted in the revised manuscript.

- Authors stated that “Stratified sampling techinique was used to select 737 mothers.” What was the criterion that you stratified and which mother you select?

Response: thank you for your meticulous observation; and sorry for what we have made an error, we have used systematic random sampling to select the study participants.

- Bivariate and multiple logistic regression analysis…. Be rewrite as bi-variable and multi-variable logistic….and it is true to for all in the body of the manscript.

Response: thank you for your suggestion; we have corrected it according to your suggestion.

- The statement “…..odds ratios at a 95% confidence interval with P-value <0.05 were considered to declare a result as statistically significant…” is redundancy that is 95% CI and p-value 0.05 are the same and writing of “p-value<0.05” is informal.

Response: thank you for your comment, we have corrected it

- “Being rural residence ……. with stillbirth” is a very long statement and did not well expressed. For instance, does rural residence a variable of level of variable? Some to others

Response: thank you for your comment, we have corrected “being residence” as residence.

- Conclusion and recommendation are not well stated and it needs amendment.

Response: thank you for your comment; we have amended it as much as possible.

**Introduction:**
-Paragraph 4 line 2-3: authors stated that “There is a problem with appropriate data on stillbirth in developing countries such as Ethiopia.” What do mean by this? I belief there is a worldwide appropriate data (EDHS-2016 and Mini EDHS-2019) and this is is not correct statement.

Response: thank you for your comment, we have corrected it. Even though we have a dearth of evidences on the prevalence and its predictors of stillbirth among delivered mothers, we have limited evidences in this study area.

-Paragraph 4 line 5-6: “The data generally available in low and middle-income countries to inform the cause of death often includes only a limited obstetric and/or reproductive history” sorry to say, I am not clear with this statement. Which data that inform the causes of death and for which death?

Response: thank you for your comment, we have amended and highlighted it in the revised manuscript.

-Lastly what is the new contribution of this manuscript to scholars and scientists worldwide? It is not well stated and showed the gap that fill full by this work.

Response: we have added the novelty of this study in the introduction section of the revised manuscript.

**Methods, results, discussion, conclusion and recommendations:**
- What do mean by SNNPR, HMIS …? It is better to state with its long form at the first time?

Response: thank you for your comment; we have spelled out when the abbreviation comes first throughout the documents.

- Why you consider Wolaita Zone among all of 14 Zones? What do mean by Zones (what is the definition of Zone in your country)?

Response: zone is a subset of region. Thus, wolaita zone is one of the densely populated zone among 14 zones in the Southern People Nation and Nationality (SNPPR) in Ethiopia.

- “According to the Wolaita zone health department…” this statement is miss placed, it is better to move somewhere as this is not indicate the study setting.

Response: thank you for your suggestion, we have corrected it.

- Your sampling method was stratified, but you calculated the sample size based on simple random sampling method. Thus, the way you calculate the sample size is wrong and better to recalculate using stratified methods.

Response: sorry for an error what we have made, we have followed systematic random sampling to select the study participants rather than stratified random sampling.

- What is/are the nature of variable(s) that you stratified your study population?

Response: sorry again, we have used systematic random sampling to select the study participants.
- A lot of grammatical errors are found in the manuscript. For instance, see this statement “Eight diploma graduate midwifery nurses as data collectors and 1 BSc midwifery and 1 health officer supervisor who speak Amharic and Wolaitegna very well were recruited.” What do mean by? Sorry, I can’t understand it!

Response: thank you for your meticulous observation, we have corrected and highlighted it in the revised manuscript.

- As far as I know “Bivariate analysis” and “bi-variable analysis” very different and I think you did not perform a bivariate analysis rather you did bi-variable. Thus, correct “bivariate and multivariate” as “bi-variable and muli-variable”

Response: thank you for your comment, we have corrected “bivariate” as bi-variable.

- You did not indicate the model that you used in this manuscript and it is better to briefly state the model in the method section.

Response: thank you for your comment, we have used binary logistic regression model for analyses.
- What is your response/outcome variable and how you define it?

Response; in this study, the outcome variable was stillbirth. We have defined stillbirth as the birth of a baby with no signs of life after 28 weeks of pregnancy recorded by health professional.

The same for your independent variables. It is better to have sub sections of variables in the study and it should explain the definition, units/levels of both outcome and explanatory variables.

Response: thank you for your suggestion; we have included it in the revised manuscript

- Some where you stated that “571 (78.8%) were grouped into age 20-34 years” and the productive age range of the country Ethiopia is from 15 to 49 years. But your sample is not representative as about 80% is from 20-34 years and these miss lead your result and conclusion. And again, the mean age range of the population is from 21.9 to 31.7 years, which indicates that the sample is not well representative of the population. By the way what do mean by SD±4.9 from “The mean age of the respondents was 26.8 (SD+ 4.9).”?

Response: thank you for your comment, by default, majority of the study participants were aged 20-34, but because of majority of the study participants from rural area, they may not know their real age.

- You stated that “The overall prevalence of stillbirth was 8.7%” does it prevalence or---? Because, (63/725)*100%=8.689% is not prevalence. Thus, first define what do mean by prevalence and you should recalculate the prevalence of stillbirth correctly otherwise your conclusion is misleading Zonal administrations!!!!!!

Response: we have corrected it in the revised manuscript

- The result section did not well state and elaborated.

Response: thank you for your comment, we have elaborated it more

- You stated that “The prevalence of stillbirth in this study showed a comparable proportion with the study conducted in the Amhara region, Ethiopia (8.5%)(10).” But Lakew [et.al](http://et.al/), 2017 stated that the proportion of stillbirth is 8.5% (218 out of 2555 samples) whereas the prevalence is 85 per 1000 total births. Thus, your comparison and definition of prevalence is totally wrong.

Response: thank you for your comment, we have amended and highlighted it in the revised manuscript.

- Consider the levels of religion that Orthodox (228) and Others (3), do you think that this frequencies are comparable and have no effect on parameter estimation? And that is why many of your predictors are not statistically significant in your model.

Response: thank you for your comment; we have not included religion in the final model

- Monthly income is lebaled as <500, 500-1500 and <= 15000, what is your evidence to label like this?

How you measure the monthly income of mothers as the majority were have no constant monthly income (housewife plus farmers =334).

Response: we have used a study done previously as reference

:

- You conclusion and recommendations are not we organized and it needs amendments.

Response: we have amended it in the revised manuscript

- Finally, Tables are not well organized, some texts are not visible and all tables needs rearrangements.

Response: we have corrected the tables as much as possible.

Reviewer #2: Dear all,
This is an important manuscript. My main point relates to how the results are being discussed. The results states that stillbirth correlates to being resident in rural areas, frequent pregnancies, and complications during pregnancy and labour. In the discussion section you tell the reader why this is the case, but without telling how this could be solved, implemented as strategies, policies, information, respectful care etc. Later in the discussion section I read recommendations and find what I search for in that section. I strongly recommend that you edit the discussion section and make the recommendations come consequently when the readers read the discussion and get the explanation of why stillbirth exists for instance in rural areas. After that the reader wish to hear your recommendation and solution of the problem, and not in a separate section further down. Now you leave the reader with a ...and what then...?? kind of feeling. It will be much better if you structure the discussion like that according to me.

You have some errors in space between words. Check that out please.

Good luck with this manuscript.

Response: thank you for your comments, we have corrected and highlighted it in the revised manuscript

Reviewer #3: Stillbirths is indeed an important problem especially in low and middle income countries (LMICs). It is therefore important to assess the prevalence and associated factors of stillbirths in these settings.
The authors aim to assess the prevalence and associated factors among deliveries attended in public hospitals of Wolaita zone, southern Ethiopia.
However, the manuscript in its current form needs some major revisions before it can be suitable for publication.

Response: thank you for your meticulous observation.

1. The methods need more detail added such as how the authors determined the number of institutions to be included in the sample. The derivation of total number of participants required was clearly shown but not the institutions.

Response: thank you for your comment; we have elaborated how the institutions were included in the revised manuscript.

2. The statistical method used to calculate the confidence interval for the proportion of stillbirths 8.7% also need to be clearly explained (was any bootstrap method used?)

Response: thank you for your question, we have used bootstrap method to calculate the confidence interval for the prevalence of stillbirth.

3. It is not clear how the initial set of predictors/independent variables were selected for inclusion in the bivariate analyses and subsequent backward elimination models. The authors may consider methods such as Directed Acyclic Graphs (DAGs) for determining the set of confounders to include in the multivariable model.

Response: thank you for your suggestion, but we have used p-value <=0.25 as cut-off point to select the predictors for multivariable model

4. Under the Data Analysis section, the authors mention that descriptive statistics were done after cleaning for inconsistencies and missing values. It would be good to mention what methods for handling missing values were employed.

Response:

5. The data analysis section also mentions that "the data was presented by texts, tables, charts,and figures". I did not come across any figures. Maybe the authors forgot to attach them with this submission.

Response: thank you for your observation, we have corrected and highlighted it in the revised manuscript.

6. The authors may consider moving the section on Data quality control to come before the Data Analysis section.

Response: thank you for your comment, we have corrected it in the revised manuscript.
