# Supplementary material for: Prevalence of stillbirth and associated factors among deliveries attended in health facilities in Southern Ethiopia
Source: PLoS One. 2022 Dec 13;17(12):e0276220. doi: 10.1371/journal.pone.0276220 (PMC9746959; doi:10.1371/journal.pone.0276220)
Supplement: Attachment — Submitted filename: Response to reviewers comment2.docx [file pone.0276220.s004.docx]

Response to reviewers comment

Reviewer #2: You have a message, you have improved but jet you need to take another round and improve the manuscript please.

**Response: thank you for your positive comment**

TITLE. I SUGGEST: Prevalence of stillbirth and its associated factors among women giving birth in healthcare facilities in southern Ethiopia
No dot in the title please

**Response: thank you for your recommendation; we have corrected and highlighted it as your suggestion.**

OBJECTIVE. I SUGGEST: To assess the prevalence and associated factors of stillbirth among women giving birth at public hospitals of Wolaita zone, southern Ethiopia

**Response: thank you for your comment; we have corrected it as your suggestion.**

RESULTS: I suggest you work with the language to make it less technical. Please replace labor with birth please. A woman give birth …“This finding showed that 8.7% of [95% CI: 6.5-10.8] the stillbirth was among women giving birth in public hospitals. Being rural residence [Adjusted OR = 2.57, 95% CI: 1.23, 5.40], number of pregnancies [Adjusted OR = 3.82, 95% CI: 1.17-12.47], complication during pregnancy and birth [Adjusted OR = 6.23, 95% CI: 2.67-14.58], history of stillbirth [Adjusted OR = 6.89, 95% CI: 2.57-13.57], type of birth [Adjusted OR = 7.13, 95% CI: 2.71-18.73] were found to be factors associated with stillbirth.”
Please choose to call them woman/women and not mothers and please do not mix from paragraph to paragraph: “The prevalence of stillbirth among mothers who gave birth i….
FORMAT: Make sure you do have space between the words: “737 mothers.A pre-tested” The format is still not fixed. It need to look like it should in the journal.
You need to re-work it again. Please take help from someone who can support you to make it look like a manuscript for submission to PLOS ONE. You have a good message to tell but you are jet not there. Please improve more.

**Response: thank you for your comment; we have improved it as much as possible.**

Reviewer #3: The authors have attended to a number of the comments raised in the first submission. However, a number of issues remain unaddressed. Generally, the manuscript still require editing as a number of statements are not very clear. For example, under Data collection process, the statement "A pretest was conducted on 5% (37 participants) of the sample size is not selected hospitals in the Wolaita zone and every possible correction was taken" This is not clear and could be completely removed as a similar statement is repeated in the Data quality control section.

**Response: thank you for your comment. We have removed it.**

On the point 3 I raised the authors have responded that all predictor variables with a p-value less than 0.25 during bivariable regression were entered into a multivariable regression. This is a fair answer but the question still remains how the variables that were entered in the bivariable regressions were selected initially. Some sort of statement justifying these or conceptual framework could be better. Identification of potential confounders can not be left to statistical models entirely.

**Response: thank you for your comment. We have included it in the revised manuscript with track change.**

The authors' response to point 4 is missing. A statement on missing data and any assumed missing data mechanism could help. I assume the the authors carried out complete case analyses which may be valid under certain missing data mechanism assumptions such as MCAR

**Response: thank you for your meticulous observation. We have included it in the revised manuscript.**
