# Supplementary material for: Prevalence of stillbirth and associated factors among deliveries attended in health facilities in Southern Ethiopia
Source: PLoS One. 2022 Dec 13;17(12):e0276220. doi: 10.1371/journal.pone.0276220 (PMC9746959; doi:10.1371/journal.pone.0276220)
Supplement: Attachment — Submitted filename: Response to reviwers R3.docx [file pone.0276220.s005.docx]

Review Comments to the Author

Please use the space provided to explain your answers to the questions above. You may also include additional comments for the author, including concerns about dual publication, research ethics, or publication ethics. (Please upload your review as an attachment if it exceeds 20,000 characters)

Reviewer #2: Prevalence of Stillbirth and associated factors among deliveries attended in Health
Facilitating Southern Ethiopia has a valuable message that I wish could be published. Still you need to work on readability and format. Revisit the instructions for authors please and make someone support you in editing the manuscript in terms of format for instance it should be the same format everywhere. In the abstract you could also help the reader to understand the important message rather than just parroting a bunch of statistical data. You know you have to help the reader to interpret your results please.

Good luck with the third revision and please improve.

Response: thank you for your comment; we have corrected and highlighted in the revised manuscript.

Reviewer #3: The authors have addressed the issues raised in the previous review but unfortunately the manuscript is still not suitable for publication in its current state. The general formatting of the manuscript requires; spaces between words, spaces after a fulstop etc
The statement by the authors stating "Missing data was managed by using MCAR" seem to suggest that MCAR is a technique for handling missing data which is not quite correct. I think stating that analyses were conducted assuming data was missing completely at random (MCAR) would be better.

Response: thank you for your valuable comment; we have corrected and highlighted in the revised manuscript.
